# Closed-Form Analysis of Stress and Deformation in Functionally Graded Multi-Layer Hyperelastic Cylinders Under Internal Pressure

**DOI:** 10.3390/ma19122642

**Published:** 2026-06-18

**Authors:** Elaheh Sarlakian, Mahdi Askari-Sedeh, Alireza Ostadrahimi, Eunsoo Choi, Majid Baniassadi, Mostafa Baghani

**Affiliations:** 1 School of Mechanical Engineering, College of Engineering, University of Tehran, Tehran 14399-57131, Iran; elahe.sarlakian@ut.ac.ir (E.S.); mahdiaskari@ut.ac.ir (M.A.-S.); m.baniassadi@ut.ac.ir (M.B.); 2 Department of Civil and Environmental Engineering, Hongik University, Seoul 04066, Republic of Korea; ostadrahimi.86@gmail.com

**Keywords:** hyperelastic, functionally graded materials, polyvinyl chloride, large deformation, Mooney–Rivlin model, thick-walled cylinder, closed-form solution, stress concentrations, internal pressure

## Abstract

This study presents a closed-form analytical solution for large-deformation pressure-induced stress and displacement fields in thick-walled, functionally graded (FG) hyperelastic polyvinyl chloride (PVC) cylinders subjected to internal pressure. The formulation inherently satisfies incompressibility—an aspect not guaranteed by standard finite element methods (FEMs)—and provides explicit expressions for all stress and deformation components. Using a Mooney–Rivlin model with an exponential–logarithmic gradation law, the study examines bi-layer and tri-layer configurations under varying property-changing scenarios. The governing equations are reduced to a single nonlinear scalar relation for the radial mapping constant, ensuring computational efficiency. Analytical predictions demonstrate excellent agreement with FEM results (errors < 1%) and recover homogeneous limits, and demonstrate that continuous gradation significantly reduces stress concentrations compared to discrete layering. The proposed model offers an efficient tool for designing pressure-resistant FG hyperelastic components for engineering applications such as pipes, hoses, biomedical devices, and protective casings.

## 1. Introduction

Exact, large-deformation predictions of pressure-driven stresses in incompressible cylindrical vessels are essential for safe and efficient design under service loads [1]. Finite-strain, calibration-based formulations outperform linearized surrogates, and analytic benchmarks remain vital because volumetric locking and stabilization methods can bias high-pressure stress predictions [2,3,4,5]. In parallel, recent advances in extreme-environment deformation measurement techniques further emphasize the need for reliable reference solutions for validation purposes [6,7]. Furthermore, as engineering demands shift toward sustainable and extreme-environment applications, the resilience of these components—defined by their ability to maintain structural integrity and functional performance under fluctuating loads—has become a paramount design objective. Recent resilience-driven frameworks for critical infrastructure emphasize that a precise understanding of stress redistribution is a prerequisite for long-term structural robustness [8], particularly when experimental stress identification relies on indirect or inverse techniques such as ultrasonic resonance-based measurements [9].

The field has recently pivoted toward complex multi-layer and functionally graded (FG) configurations to optimize performance. Recent exact and semi-analytical vessel studies show peak stresses are highly sensitive to constitutive model and boundary conditions, driving the need for parameter-transparent predictions [10,11]. Biomedical applications heighten these requirements, while studies of graded elastomers demonstrate that smooth through-thickness property variation reshapes pressure-driven stress profiles, motivating exact treatments of gradation effects. Specifically, 2025 investigations into multi-layer pressurized cylinders have provided exact solutions for shrink-fit interactions, yet they often rely on piecewise-constant descriptions that can obscure the redistribution of stresses provided by truly continuous gradation [12,13].

Despite established large-deformation vessel studies, a clear gap remains: an exact hyperelastic formulation that predicts pressure–stress fields in continuously graded, multi-layer hyperelastic cylinders subjected to internal pressure. Analytical (ANL) and semi-analytical multi-layer treatments provide closed-form relations for layered vessels, but their piecewise-constant property descriptions can obscure how truly continuous gradation redistributes pressure-driven stresses and masks gradation-specific effects [14,15,16]. Semi-analytical finite-length and three-dimensional treatments demonstrate the importance of strictly enforcing boundary conditions and incompressibility to control peak stresses, yet most do not couple those enforcement details to a continuous through-thickness gradation [17,18,19].

A significant challenge remains in the mathematical coupling of continuous gradation with exact kinematic enforcement. Independent analyses of FG hyperelastic cylinders show that smooth radial variation in constitutive parameters reshapes radial and hoop stress profiles, motivating continuous gradation laws rather than stepwise layering when accurate, parameter-transparent predictions are required [20]. Benchmark ring and tube studies further show that abrupt stiffness jumps introduce artificial stress discontinuities that complicate comparisons with graded limits, underscoring the need for an exact formulation that preserves incompressibility while eliminating interface artifacts when passing to FG descriptions [21]. Together, these works set a clear target: an exact, closed-form FG finite-strain theory for internally pressurized cylinders that yields parameter-transparent pressure–stress predictions and recovers multi-layer and homogeneous results as piecewise-constant and zero-gradient limits, respectively [14,22].

Assuming axisymmetry with plane strain fixes the kinematics and allows exact enforcement of incompressibility. From a numerical perspective, the necessity of analytical benchmarks is reinforced by the persistent challenges of the incompressible limit in finite element method (FEM) software(the FEM calculations were performed using COMSOL Multiphysics^®^ 6.3.0.290). Recent mixed and stabilized formulations demonstrate that substituting an artificially large bulk modulus remains prone to volumetric locking and pressure bias, whereas mixed displacement–pressure schemes preserve reliable stress predictions at finite strain. State-of-the-art 2025 research continues to propose new four-field mixed formulations and unified three-dimensional elements to address these stability issues in nearly and fully incompressible solids [23,24]. Benchmarks for large-strain hyperelastic elements further show that predicted stress peaks and through-thickness gradients depend critically on how incompressibility and boundary conditions are enforced as well as on the chosen strain-energy form (e.g., Neo-Hookean versus Mooney–Rivlin), reinforcing the need to specify the constitutive class explicitly and to audit enforcement details [25]. In numerical practice, contact algorithms or artificial prescriptions (for example, virtual thermal strains used to mimic assembly loads) can conflate loading kinematics with solution procedures and thereby obscure parameter transparency at interfaces [26,27]. In this study we instead prescribe boundary tractions and geometric constraints directly, enforce exact incompressibility, and require continuity of displacement and stress across layers. Functional gradation is introduced as a smooth radial variation in the stored-energy parameters; recent analyses of graded tubes under coupled inflation–torsion illustrate how continuous laws reshape hoop and axial responses but do not address the combined effect of continuous gradation and explicit boundary-traction prescriptions [15,28,29]. These considerations define our modeling choices: axisymmetric, plane-strain, incompressible hyperelasticity with direct enforcement of incompressibility and a smooth FG law tied to the stored-energy parameters [23,24,25,26].

Radial equilibrium together with exact incompressibility reduces the boundary-value problem to a single scalar condition for an integration constant that is fixed by the prescribed radial tractions. Once that constant is found, the radial mapping and closed-form expressions for radial displacement and for all stress components follow algebraically. Multi-layer analytical treatments make this scalar structure explicit for piecewise-constant parameter distributions [30]. Analytical solutions for FG cylinders (including studies of inflation and coupled loading) preserve the same scalar organization after replacing piecewise constants with smooth radial functions, although many such works treat different loading modes or simplifying assumptions and do not emphasize a traction-based scalar closure for graded materials [31]. Other graded-cylinder analyses likewise exploit the scalar reduction but focus on alternate loadings or numerical strategies rather than on an explicit, closed-form traction closure [32,33]. By contrast, finite element approximations generally require mixed or stabilized formulations to control volumetric locking and spurious pressure oscillations; their accuracy depends sensitively on how near-incompressibility and boundary data are enforced, and an explicit scalar closure typically emerges only through post-processing rather than directly from the discrete formulation [34,35]. This scalar framework yields an analytically tractable basis for quantifying how continuous gradation modifies stress distributions and for producing design-relevant, parameter-transparent maps [31,32,34].

We present an exact, closed-form solution for the large-deformation radial mapping, displacements, and full stress field of a thick-walled, incompressible, FG multi-layer hyperelastic cylinder under internal pressure. The formulation enforces incompressibility exactly, admits general smooth through-thickness gradation laws (here illustrated with a Mooney–Rivlin model and an exponential–logarithmic gradation), and reduces the boundary-value problem to a single scalar closure whose solution yields algebraic expressions for radial, hoop and axial stresses. The theory recovers piecewise-constant multi-layer and homogeneous limits and is applied to representative bi-layer and tri-layer PVC [36] cases with two gradation variants. Analytical predictions are benchmarked against mixed/hybrid finite element solutions. Parametric studies quantify how grading strength and layer contrasts govern stress smoothing and peak magnitudes, producing directly usable maps for the design of graded elastomer pressure vessels. The closed-form base state also enables straightforward extensions (incremental stability, non-axisymmetric or finite-length numerical studies, and thermo-mechanical coupling).

## 2. Materials and Methods

### 2.1. Materials

#### Material Model and Properties

In continuum mechanics, hyperelastic materials are characterized by an energy density function. In this study, the Mooney–Rivlin model was employed, which consists of two material parameters, denoted as *C*_10_ and *C*_01_.

To incorporate FG distribution of these material constants, two cases were studied: one with increasing and the other with decreasing material properties as the radius increases. Multiplication factors (MFs) of 1.2 and 0.8 were used to scale the properties to their maximum and minimum values from the base values. The main values, which maximization and minimization were performed on, were chosen by the properties belonging to PVC, which are reported in Table 1.

In each case study, regardless of the number of layers and whether the material properties increase or decrease with the radius, two values were defined for each parameter within a layer. For instance, ${C_{10}}_{max}$ and ${C_{10}}_{min}$ refer to the maximum and minimum values of $C_{10}$.

Within the layer. Similarly, $C_{01,max}$ and $C_{01,min}$ refer to the maximum and minimum values of $C_{01}$ within the same layer. The $C_{10}$ and $C_{01}$ properties of PVC were utilized for the first layer.

Additionally, in this study, two cases of bi-layer and multi-layer cylinders were analyzed. In both cases, the material properties of the inner layer were higher than those of the outer layers. This is because the inner layer is in direct contact with the location where the pressure is applied, and required to be stronger. In the bi-layer cylinder, the properties of the second layer were 50% lower than those of the first layer. The material properties of the bi-layer cylinder with two MFs of 0.8 and 1.2 are provided in Table 2 and Table 3, respectively.

In the multi-layer cylinder, the properties of the second and third layers were 30% and 60% lower than those of the first layer, respectively. The material properties of the multi-layer cylinder, with two MFs of 0.8 and 1.2, are provided in Table 4 and Table 5, respectively.

### 2.2. Methods

#### 2.2.1. Analytical Solution

**Figure 1:**
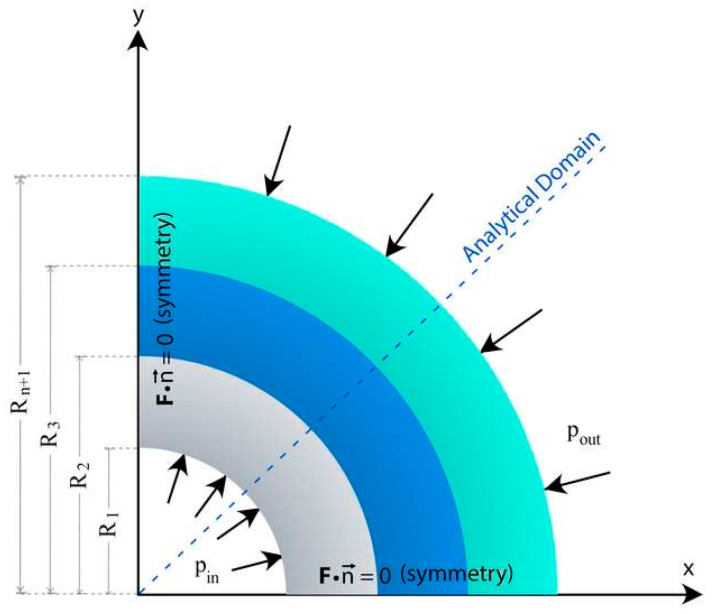
The schematic of a multi-layer cylinder subjected to inner ($p_{in}$) and outer ($p_{out}$) radial pressure; the different colors serve solely to distinguish the individual layers and do not represent field quantities or contour values.

Figure 1 illustrates the schematic of a thick, long, multi-layer cylinder under radial pressure. Due to the infinite length of the cylinder, plain strain assumption is made to simplify the analytical approach, as the longitudinal variation in stress and displacement fields can be neglected. A distinct material is assigned to each layer of the cylinder, and the thickness can vary from one layer to another. However, in this study, equal thickness is assumed for all layers. Given the theory of hyperelasticity, which is a branch of nonlinear mechanics, the proposed analytical solution must be capable of capturing the large deformation and inflation caused by the applied radial pressure.

The inner and outer radial pressures are denoted as $p_{in}$ and $p_{out}$, respectively. Each layer consists of two radii: the inner and outer radii, denoted as $R_{in}\;and\;R_{out}$, respectively. The radii $R_{1},R_{2},R_{3},\;and\;R_{n+1}$ correspond to the inner radius of the first, second, third, and the last layer, respectively.

The general form of the deformation gradient in the cylindrical coordinate system [39] is as follows:

(1) $$\;F=\left[\begin{array}{ccc} \frac{\partial r}{\partial R} & \frac{1}{R}\frac{\partial r}{\partial \Theta } & \frac{\partial r}{\partial Z}\\ r\frac{\partial \theta }{\partial R} & \frac{r}{R}\frac{\partial \theta }{\partial \Theta } & r\frac{\partial \theta }{\partial Z}\\ \frac{\partial z}{\partial R} & \frac{1}{R}\frac{\partial z}{\partial \Theta } & \frac{\partial z}{\partial Z} \end{array}\right].$$

In this problem, all fields depend only on the reference radial coordinate, so the deformation is represented by the monotone radial mapping [14],

(2) $$r=r\left(R\right),\;\theta =\Theta ,\;z=Z.$$

where r and R are the current and reference radii. Using this assumption, the deformation gradient tensor is defined as:

(3) $$F=\left[\begin{array}{ccc} \frac{\partial r}{\partial R} & 0 & 0\\ 0 & \frac{r}{R} & 0\\ 0 & 0 & 1 \end{array}\right].$$

The left Cauchy–Green deformation tensor is given as follows [40]:

(4) $$b=FF^{T}=\left[\begin{array}{ccc} {\left(\frac{\partial r}{\partial R}\right)}^{2} & 0 & 0\\ 0 & {\left(\frac{r}{R}\right)}^{2} & 0\\ 0 & 0 & 1 \end{array}\right].$$

The first three principal invariants of the left Cauchy–Green deformation tensors are defined as $I_{1},I_{2},\;and\;I_{3}$ as follows [40]:

(5) $$I_{1}=tr\left(b\right)={\left(\frac{\partial r}{\partial R}\right)}^{2}+{\left(\frac{r}{R}\right)}^{2}+1,$$

(6) $$I_{2}=\frac{1}{2}\left[{\left(trb\right)}^{2}-tr(b^{2})\right]=\left[{\left(\frac{\partial r}{\partial R}\right)}^{2}{\left(\frac{r}{R}\right)}^{2}+{\left(\frac{\partial r}{\partial R}\right)}^{2}+{\left(\frac{r}{R}\right)}^{2}\right],$$

(7) $$I_{3}=\left|b\right|={\left|F\right|}^{2}=J^{2}=1.$$

Exact incompressibility closes the kinematics through the radial identity as follows:

(8) $$J=1\to \left(\frac{\partial r}{\partial R}\right)\left(\frac{r}{R}\right)=1\to r\frac{\partial r}{\partial R}=R.$$

The displacement field is defined as:

(9) u=r−R.

Integration of the incompressibility relation yields an explicit primitive for mapping,

(10) $$r^{2}(R)=R^{2}+c.$$

where c is the integration constant.

To impose the continuity condition on the radial deformation of the successive layers, the following steps were taken:

(11) $$at\;R=R_{n+1}\to u_{r,n}=u_{r,n+1}.$$

u was substituted from Equation (9) into Equation (11):

(12) $$\sqrt{R_{n+1}^{2}+c_{n}}-R_{n+1}=\sqrt{R_{n+1}^{2}+c_{n+1}}-R_{n+1}.$$

which concludes:

(13) $$c_{n}=c_{n+1};\;n=1,\;2,\;\ldots ,\;n-1.$$

This implies that $c_{n}$ does not change from one layer to another.

For an incompressible hyperelastic cylinder, the Mooney–Rivlin model [41] is defined as:

(14) $$\psi =C_{10}\;\left(I_{1}-3\right)+\;C_{01}\;\left(I_{2}-3\right)$$

where $C_{10}\;and\;C_{01}$ are the material constants.

To obtain an expression for the different components of Cauchy stress, the second Piola stress was first defined as [40]:

(15) $$S=2\frac{\partial \psi }{\partial C}=2\left[\frac{\partial \psi }{\partial I_{1}}\frac{\partial I_{1}}{\partial C}+\frac{\partial \psi }{\partial I_{2}}\frac{\partial I_{2}}{\partial C}+\frac{\partial \psi }{\partial I_{3}}\frac{\partial I_{3}}{\partial C}\right]$$

The derivatives of the principal invariants with respect to the right Cauchy–Green deformation tensor can be calculated as [40]:

(16) $$\;\frac{\partial I_{1}}{\partial C}=I;\;\frac{\partial I_{2}}{\partial C}=I_{1}I-C;\;\frac{\partial I_{3}}{\partial C}=I_{3}C^{-1}.\;$$

By substituting Equation (16) into Equation (15), the expression for the second Piola stress tensor [42] is as follows:

(17) $$S=2\left[\frac{\partial \psi }{\partial I_{1}}I+\frac{\partial \psi }{\partial I_{2}}\left(I_{1}I-C\right)+\frac{\partial \psi }{\partial I_{3}}\left(I_{3}C^{-1}\right)\right].$$

The Cauchy stress formula can be written using its relation with the second Piola stress as follows [40]:

(18) $$\begin{array}{l} \sigma =\frac{1}{J}FSF^{T}=\frac{2}{J}F\left[\frac{\partial \psi }{\partial I_{1}}I+\frac{\partial \psi }{\partial I_{2}}\left(I_{1}I-C\right)+\frac{\partial \psi }{\partial I_{3}}\left(I_{3}C^{-1}\right)\right]F^{T}\\ \;=\frac{2}{\sqrt{I_{3}}}\left[\frac{\partial \psi }{\partial I_{1}}b+\frac{\partial \psi }{\partial I_{2}}\left(I_{1}B-{F\left(F^{T}F\right)F}^{T}\right)+\frac{\partial \psi }{\partial I_{3}}\left(I_{3}F\left(F^{-1}F^{-T}\right)F^{T}\right)\right]\\ \;=\frac{2}{\sqrt{I_{3}}}\left[\frac{\partial \psi }{\partial I_{1}}b+\frac{\partial \psi }{\partial I_{2}}\left(I_{1}b-b^{2}\right)+\frac{\partial \psi }{\partial I_{3}}\left(I_{3}I\right)\right]. \end{array}$$

The index notation for Equation (18) is:

(19) $$\sigma_{ij}=\frac{2}{\sqrt{I_{3}}}\left[\left(\frac{\partial \psi }{\partial I_{1}}+I_{1}\frac{\partial \psi }{\partial I_{2}}\right)b_{ij}-\frac{\partial \psi }{\partial I_{2}}\sum_{m=1}^{3}b_{im}b_{mj}\right]+2\sqrt{I_{3}}\frac{\partial \psi }{\partial I_{3}}\delta_{ij}.$$

An incompressible material is only deformed when subjected to isochoric stress, which is denoted as ${\bar{\sigma }}_{ij}.$ Incompressibility constraint does not allow the material to change its volume, so volumetric stress, which is denoted as −p, plays no role in material deformation.

The total stress, which is the summation of isochoric and volumetric stress, is defined as follows:

(20) $$\sigma_{ij}={\bar{\sigma }}_{ij}+\left(-p\right)\delta_{ij}.\;$$

where $\delta_{ij}$ is the kronecker delta, defined as [40]:

(21) $$\delta_{ij}=\left\{\begin{array}{c} 0\\ 1 \end{array}\begin{array}{c} \;if\;i\neq j\\ \;if\;i=j \end{array}\right.$$

The principal stress is defined as:

(22) $$\sigma_{i}\equiv \sigma_{ii}={\bar{\sigma }}_{i}-p$$

Equation (18) is simplified by using Equation (22), $I_{3}=1,\;and\;\frac{\partial \psi }{\partial I_{3}}=0$, as:

(23) $$\sigma_{i}=2\left[\left(\frac{\partial \psi }{\partial I_{1}}+I_{1}\frac{\partial \psi }{\partial I_{2}}\right)b_{ii}-\frac{\partial \psi }{\partial I_{2}}\sum_{m=1}^{3}b_{im}b_{mi}\right]-p$$

The radial, tangential, and axial stress components, denoted as $\sigma_{r},\sigma_{\theta },\;and\;\sigma_{z}$, respectively, are defined using Equation (23):

(24) $$\begin{array}{l} \sigma_{r}=2\frac{\partial \psi }{\partial I_{1}}b_{11}+2\frac{\partial \psi }{\partial I_{2}}\left(I_{1}b_{11}-b_{11}^{2}\right)-p\\ \;=2C_{10}\left(\frac{R^{2}}{r^{2}}\right)+2C_{01}\left(\left(\left(\frac{R^{2}}{r^{2}}\right)+\left(\frac{r^{2}}{R^{2}}\right)+1\right)\left(\frac{R^{2}}{r^{2}}\right)-\left(\frac{R^{4}}{r^{4}}\right)\right)-p\\ \;\overset{1+\left(\frac{R^{2}}{r^{2}}\right)=-\frac{r^{2}}{R^{2}}}{\to }\;\sigma_{r}=2C_{10}{\left(\frac{R}{r}\right)}^{2}-2C_{01}{\left(\frac{r}{R}\right)}^{2}-p \end{array}$$

(25) $$\begin{array}{l} \sigma_{\theta }=2\frac{\partial \psi }{\partial I_{1}}b_{22}+2\frac{\partial \psi }{\partial I_{2}}\left(I_{1}b_{22}-b_{22}^{2}\right)-p\\ \;=2C_{10}\left(\frac{r^{2}}{R^{2}}\right)+2C_{01}\left(\left(\left(\frac{R^{2}}{r^{2}}\right)+\left(\frac{r^{2}}{R^{2}}\right)+1\right)\left(\frac{r^{2}}{R^{2}}\right)-\left(\frac{r^{4}}{R^{4}}\right)\right)-p\\ \;\overset{1+\left(\frac{r^{2}}{R^{2}}\right)=-\frac{R^{2}}{r^{2}}}{\to }{\;\sigma }_{\theta }=2C_{10}{\left(\frac{r}{R}\right)}^{2}-2C_{01}{\left(\frac{R}{r}\right)}^{2}-p \end{array}$$

(26) $$\begin{array}{l} \sigma_{z}=2\frac{\partial \psi }{\partial I_{1}}b_{33}+2\frac{\partial \psi }{\partial I_{2}}\left(I_{1}b_{33}-b_{33}^{2}\right)-p=2\frac{\partial \psi }{\partial I_{1}}-2\frac{\partial \psi }{\partial I_{2}}-p\\ \;=\sigma_{r}-2\frac{\partial \psi }{\partial I_{1}}\left[{\left(\frac{R}{r}\right)}^{2}-1\right]+2\frac{\partial \psi }{\partial I_{2}}\left[{\left(\frac{r}{R}\right)}^{2}-1\right]-p\\ \;=\sigma_{r}+2\left(C_{01}\frac{c}{R^{2}}+C_{10}\frac{c}{R^{2}+c}\right)-p \end{array}$$

In polar coordinates, in the absence of body forces, the radial stress equilibrium is defined as follows [43]:

(27) $$\frac{d\sigma_{r}}{dr}+\frac{\sigma_{r}-\sigma_{\theta }}{r}=0$$

Now, the expression related to $\sigma_{r}$ and $\sigma_{\theta }$ can be substituted from Equation (24) and Equation (25), respectively, into Equation (27):

(28) $$\frac{d\sigma_{r}}{dr}=-\frac{2}{r}\left(C_{10}+C_{01}\right)\left[{\left(\frac{R}{r}\right)}^{2}-{\left(\frac{r}{R}\right)}^{2}\right]$$

Using Equation (8), Equation (28) becomes:

(29) $$\frac{d\sigma_{r}}{dR}=-\frac{2R}{r^{2}}\left(C_{10}+C_{01}\right)\left[{\left(\frac{R}{r}\right)}^{2}-{\left(\frac{r}{R}\right)}^{2}\right]$$

Then, integration is applied to both sides of Equation (29):

(30) $$\sigma_{r}=\int_{R_{n}}^{R}\frac{d\sigma_{r}}{dR}\;dR=\int_{R_{n}}^{R}\left[-\frac{2R}{r^{2}}\left(C_{10}+C_{01}\right)\left[{\left(\frac{R}{r}\right)}^{2}-{\left(\frac{r}{R}\right)}^{2}\right]\right]\;dR$$

By using Equation (10), Equation (30) is rewritten as follows:

(31) $$\sigma_{r}=\int_{R_{n}}^{R}\frac{d\sigma_{r}}{dR}\;dR=\int_{R_{n}}^{R}\left[-2\left(C_{10}+C_{01}\right)\left[\frac{R^{3}}{{\left(R^{2}+c_{n}\right)}^{2}}-\frac{1}{R}\right]\right]\;dR$$

Equation (31) is suitable for problems where material properties are assumed to be homogenous. To generalize the solution for heterogeneous material distributions, an FG approach is employed for the Mooney–Rivlin material parameters, *C*_10_ and *C*_01_.

To account for the FG distribution, an exponential–logarithmic distribution form of $C_{10}$ and $C_{01}$ is defined in Equation (32) and Equation (33), respectively:

(32) $$C_{10}(R)={C_{10}}_{min}exp\left(\frac{R-R_{out}}{R_{out}-R_{in}}\ln\left(\frac{{C_{10}}_{max}}{{C_{10}}_{min}}\right)\right).$$

(33) $$C_{01}(R)={C_{01}}_{min}exp\left(\frac{R-R_{out}}{R_{out}-R_{in}}\ln\left(\frac{{C_{01}}_{max}}{{C_{01}}_{min}}\right)\right).$$

The homogeneous limit is recovered when $\frac{{C_{01}}_{max}}{{C_{01}}_{min}}=1\;and\;\frac{{C_{10}}_{max}}{{C_{10}}_{min}}=1.$

The expressions for $C_{10}\left(R\right)\;and\;C_{01}(R)$ are required to be substituted into Equation (31) to employ the integration process. To facilitate integration, the Taylor expansion is used for the exponential part of Equations (32) and (33), expanded up to four terms. The expansion is used as follows:

(34) $$\begin{array}{l} C_{10}(R)={C_{10}}_{min}(1+\left(\frac{R-R_{out}}{R_{out}-R_{in}}\ln\left(\frac{{C_{10}}_{max}}{{C_{10}}_{min}}\right)\right)+\frac{1}{2}{\left(\frac{R-R_{out}}{R_{out}-R_{in}}\ln\left(\frac{{C_{10}}_{max}}{{C_{10}}_{min}}\right)\right)}^{2}\\ \;+\frac{1}{6}{\left(\frac{R-R_{out}}{R_{out}-R_{in}}\ln\left(\frac{{C_{10}}_{max}}{{C_{10}}_{min}}\right)\right)}^{3}). \end{array}$$

(35) $$\begin{array}{l} C_{01}(R)={C_{01}}_{min}(1+\left(\frac{R-R_{out}}{R_{out}-R_{in}}\ln\left(\frac{{C_{01}}_{max}}{{C_{01}}_{min}}\right)\right)+\frac{1}{2}{\left(\frac{R-R_{out}}{R_{out}-R_{in}}\ln\left(\frac{{C_{01}}_{max}}{{C_{01}}_{min}}\right)\right)}^{2}\\ \;+\frac{1}{6}{\left(\frac{R-R_{out}}{R_{out}-R_{in}}\ln\left(\frac{{C_{01}}_{max}}{{C_{01}}_{min}}\right)\right)}^{3}). \end{array}$$

Now, $C_{10}\left(R\right)\;and\;C_{01}(R)$ are substituted into Equation (31):

(36) $$\begin{array}{l} \sigma_{r}=\int_{R_{n}}^{R}[-2(({C_{10}}_{min}(1+(\frac{R-R_{out}}{R_{out}-R_{in}}\ln\left(\frac{{C_{10}}_{max}}{{C_{10}}_{min}}\right))+\frac{1}{2}{\left(\frac{R-R_{out}}{R_{out}-R_{in}}\ln\left(\frac{{C_{10}}_{max}}{{C_{10}}_{min}}\right)\right)}^{2}\\ \;+\frac{1}{6}{\left(\frac{R-R_{out}}{R_{out}-R_{in}}\ln\left(\frac{{C_{10}}_{max}}{{C_{10}}_{min}}\right)\right)}^{3}))\\ \;+({C_{01}}_{min}(1+(\frac{R-R_{out}}{R_{out}-R_{in}}\ln\left(\frac{{C_{01}}_{max}}{{C_{01}}_{min}}\right))\\ \;+\frac{1}{2}{\left(\frac{R-R_{out}}{R_{out}-R_{in}}\ln\left(\frac{{C_{01}}_{max}}{{C_{01}}_{min}}\right)\right)}^{2}\\ \;+\frac{1}{6}{\left(\frac{R-R_{out}}{R_{out}-R_{in}}\ln\left(\frac{{C_{01}}_{max}}{{C_{01}}_{min}}\right)\right)}^{3})))[\frac{R^{3}}{{\left(R^{2}+c_{n}\right)}^{2}}-\frac{1}{R}]]dR-p_{n}. \end{array}$$

where $p_{n}$ denotes the pressure applied on the radius of $R_{n}$. The above integral requires a long-hand solution. The final closed-form solution is reported in Appendix A.

By considering ${R=R}_{n+1}$, Equation (36) becomes as follows:

(37) $$\begin{array}{l} p_{n}-p_{n+1}\;=\int_{R_{n}}^{R}[-2(({C_{10}}_{min}(1+(\frac{R-R_{out}}{R_{out}-R_{in}}\ln\left(\frac{{C_{10}}_{max}}{{C_{10}}_{min}}\right))\\ \;+\frac{1}{2}{\left(\frac{R-R_{out}}{R_{out}-R_{in}}\ln\left(\frac{{C_{10}}_{max}}{{C_{10}}_{min}}\right)\right)}^{2}+\frac{1}{6}{\left(\frac{R-R_{out}}{R_{out}-R_{in}}\ln\left(\frac{{C_{10}}_{max}}{{C_{10}}_{min}}\right)\right)}^{3}))\\ \;+({C_{01}}_{min}(1+(\frac{R-R_{out}}{R_{out}-R_{in}}\ln\left(\frac{{C_{01}}_{max}}{{C_{01}}_{min}}\right))\\ \;+\frac{1}{2}{\left(\frac{R-R_{out}}{R_{out}-R_{in}}\ln\left(\frac{{C_{01}}_{max}}{{C_{01}}_{min}}\right)\right)}^{2}\\ \;+\frac{1}{6}{\left(\frac{R-R_{out}}{R_{out}-R_{in}}\ln\left(\frac{{C_{01}}_{max}}{{C_{01}}_{min}}\right)\right)}^{3})))[\frac{R^{3}}{{\left(R^{2}+c_{n}\right)}^{2}}-\frac{1}{R}]]\;dR. \end{array}$$

where $p_{n+1}$ denotes the pressure applied on the radius of $R_{n+1}$, which is equal to $\sigma_{n+1}$.

To obtain $c_{n}$, which is the same for all layers within the cylinder, Equation (37) must be solved successively, as shown in Equation (38):

(38) $$\begin{array}{l} p_{in}-p_{out}=\sum_{m=1}^{m=n}p_{m}-p_{m+1}\\ \;=2\sum_{m=1}^{n}(\int_{R_{n}}^{R}[-2(({C_{10}}_{min,m}(1+(\frac{R-R_{out,m}}{R_{out,m}-R_{in,m}}\ln\left(\frac{{C_{10}}_{max,m}}{{C_{10}}_{min,m}}\right))\\ \;+\frac{1}{2}{\left(\frac{R-R_{out,m}}{R_{out,m}-R_{in,m}}\ln\left(\frac{{C_{10}}_{max,m}}{{C_{10}}_{min,m}}\right)\right)}^{2}\\ \;+\frac{1}{6}{\left(\frac{R-R_{out,m}}{R_{out,m}-R_{in,m}}\ln\left(\frac{{C_{10}}_{max,m}}{{C_{10}}_{min,m}}\right)\right)}^{3}))\\ \;+({C_{01}}_{min}(1+(\frac{R-R_{out,m}}{R_{out,m}-R_{in,m}}\ln\left(\frac{{C_{01}}_{max,m}}{{C_{01}}_{min,m}}\right))\\ \;+\frac{1}{2}{\left(\frac{R-R_{out,m}}{R_{out,m}-R_{in,m}}\ln\left(\frac{{C_{01}}_{max,m}}{{C_{01}}_{min,m}}\right)\right)}^{2}\\ \;+\frac{1}{6}{\left(\frac{R-R_{out,m}}{R_{out,m}-R_{in,m}}\ln\left(\frac{{C_{01}}_{max,m}}{{C_{01}}_{min,m}}\right)\right)}^{3})))\left[\frac{R^{3}}{{\left(R^{2}+c_{n}\right)}^{2}}-\frac{1}{R}\right]]\;dR) \end{array}$$

where ${C_{10}}_{min,m},{C_{10}}_{max,m},{C_{01}}_{min,m}\;and\;{C_{01}}_{max,m}$ are the minimum and maximum values of $C_{10}$ and $C_{01}$ in the m-th layer, respectively. $R_{in,m}\;and\;R_{out,m}$ denote the inner and outer radii of the m-th layer. Also, to implement the traction free of outermost surface, $p_{out}$ is considered zero. The nonlinear scalar closure in Equation (37) is solved for the mapping constant c using a Newton-type root-finding algorithm (via MATLAB R2024b’s fsolve), with convergence established when the residual falls below a relative function tolerance of $10^{-6}$.

By starting from the outer radius and solving Equation (37) successively, the pressure applied on each boundary radius is obtained.

After solving Equation (36), the tangential and axial stresses are calculated in Equation (39) and Equation (40), respectively:

(39) $$\sigma_{\theta }=\sigma_{r}+2{\left(C_{10}+C_{01}\right)}_{k}\left(\frac{R^{2}+c_{n}}{R^{2}}-\frac{R^{2}}{R^{2}+c_{n}}\right).$$

(40) $$\sigma_{z}=\sigma_{r}+2\left[{\left(C_{10}\right)}_{k}\frac{c_{n}}{R^{2}}+{\left(C_{01}\right)}_{k}\frac{c_{1}}{R^{2}+c_{n}}\right].$$

The generality of the present formulation allows for the exact recovery of the classical homogeneous solution for an incompressible hyperelastic cylinder pressurized on both surfaces as a limiting case [35]. By enforcing constant material parameters across the wall thickness and prescribing a non-zero outer pressure ($p_{out}\neq 0$), the governing equations reduce to the established benchmarks for doubly pressurized Mooney–Rivlin cylinders.

#### 2.2.2. Finite Element Solution

To verify the analytical solution, finite element analysis of bi-layer and multi-layer cylinders subjected to internal pressure was carried out using COMSOL Multiphysics version 6.3.0.290 software. To capture hyperelasticity and large deformation, a Mooney–Rivlin material model was used. This model includes two material parameters, denoted as C_10_ and C_01_. A quadrilateral element family was used with a structured mapped mesh (aligned with the radial direction) to accurately resolve through-thickness stress gradients and discontinuities at the layer interfaces. For incompressibility, a mixed (hybrid) displacement–pressure (u–p) formulation was adopted to prevent volumetric locking. The mesh was generated using COMSOL’s “Extremely Fine” setting, with a maximum element size of 0.11 mm.

##### Geometry

**Figure 2:**
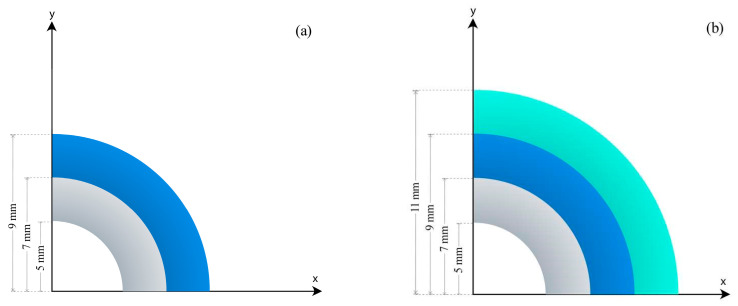
(**a**) the bi-layer cylinder; (**b**) the multi-layer cylinder; the different colors serve solely to distinguish the individual layers and do not represent field quantities or contour values.

One quarter of each cylinder was modeled to reduce the simulation runtime. The geometries of the bi-layer and multi-layer cylinders are illustrated in Figure 2a and Figure 2b, respectively. No gap or interference was assumed between the layers. The outer layer is highlighted in blue in each case.

The inner and outer radii of each layer for the bi-layer and multi-layer cases are presented in Table 6 and Table 7, respectively.

##### Boundary Conditions

To prevent any undesirable movement of the cylinders, two displacement-type boundary conditions were imposed. The vertical displacements of radii that lie horizontally in parallel to the *x*-axis were prescribed as zero, and the horizontal displacements of radii that lie vertically in parallel to the *y*-axis were prescribed as zero. Radial pressure is directly applied to the inner radius of the cylinder. To ensure symmetric results, a symmetry boundary condition was defined for the radii that are parallel to the *x*-axis and *y*-axis.

## 3. Results

### 3.1. The Bi-Layer Cylinder

#### 3.1.1. Increasing Material Properties with Increasing Radius

**Figure 3:**
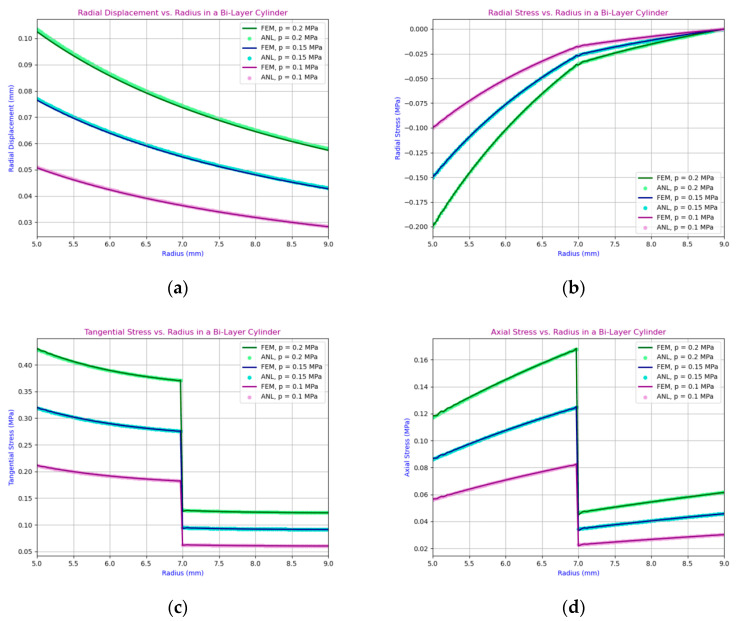
Radial displacement and stress distributions of the bi-layer cylinder in the case of increasing material properties with increasing radius: (**a**) radial displacement; (**b**) radial stress; (**c**) tangential stress; (**d**) axial stress.

The radial displacement and stress distributions of the bi-layer cylinder in the case of increasing material properties with increasing radius are shown in Figure 3a, Figure 3b, Figure 3c and Figure 3d, respectively.

The error between the analytical and finite element solutions in the bi-layer cylinder, in the case of increasing material properties with increasing radius, is reported in Table 8.

#### 3.1.2. Decreasing Material Properties with Increasing Radius

**Figure 4:**
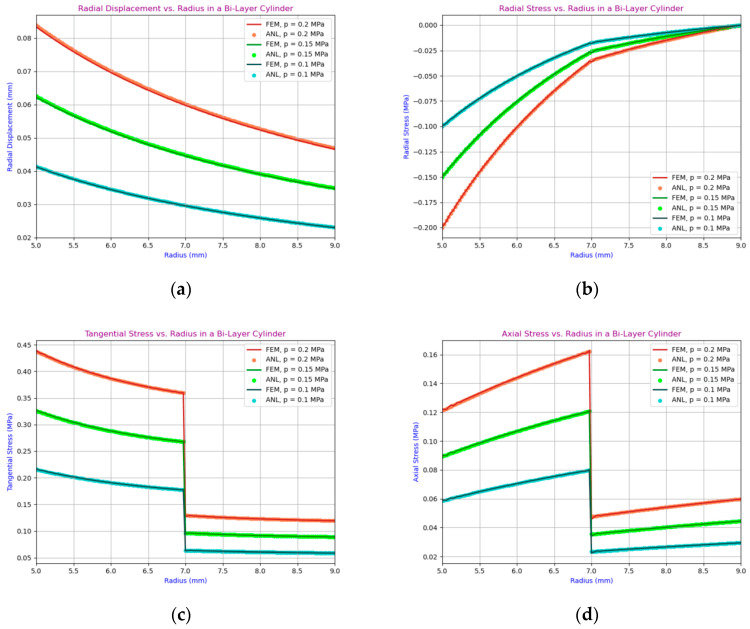
The radial displacement and stress distributions of the bi-layer cylinder in the case of decreasing material properties with increasing radius: (**a**) radial displacement; (**b**) radial stress; (**c**) tangential stress; (**d**) axial stress.

The radial displacement and stress distributions of the bi-layer cylinder in the case of decreasing material properties with increasing radius are shown in Figure 4a, Figure 4b, Figure 4c and Figure 4d, respectively.

The error between the analytical and finite element solutions in the bi-layer cylinder, in the case of decreasing material properties with increasing radius, is reported in Table 9.

### 3.2. The Multi-Layer Cylinder

#### 3.2.1. Increasing Material Properties with Increasing Radius

**Figure 5:**
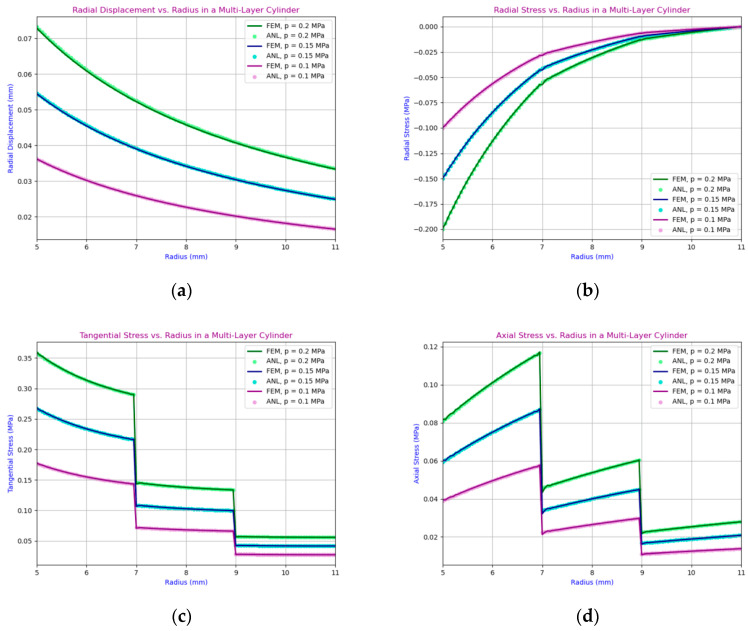
The radial displacement and stress distributions of the multi-layer cylinder in the case of increasing material properties with increasing radius: (**a**) radial displacement; (**b**) radial stress; (**c**) tangential stress; (**d**) axial stress.

The radial displacement and stress distributions of the multi-layer cylinder in the case of increasing material properties with increasing radius are shown in Figure 5a, Figure 5b, Figure 5c and Figure 5d, respectively.

The error between the analytical and finite element solutions in the multi-layer cylinder, in the case of increasing material properties with increasing radius, is reported in Table 10.

#### 3.2.2. Decreasing Material Properties with Increasing Radius

**Figure 6:**
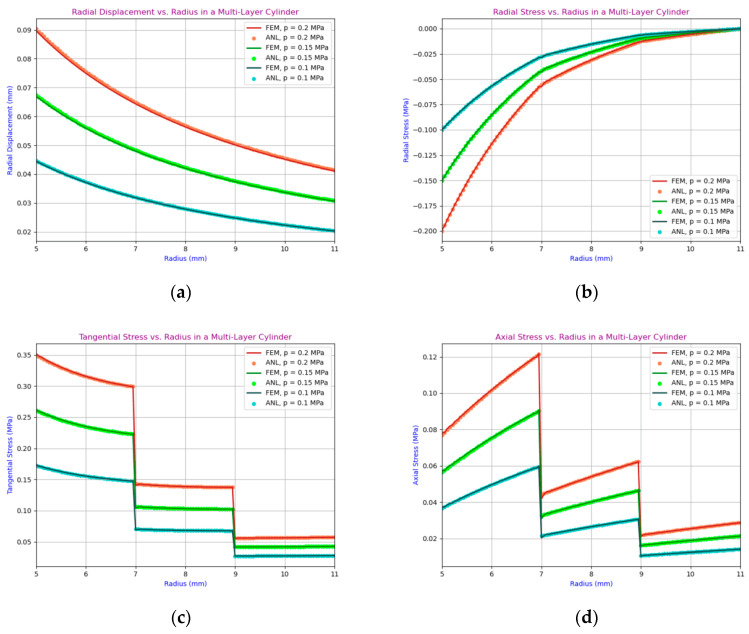
The radial displacement and stress distributions of the multi-layer cylinder in the case of decreasing material properties with increasing radius: (**a**) radial displacement; (**b**) radial stress; (**c**) tangential stress; (**d**) axial stress.

The radial displacement and stress distributions of the multi-layer cylinder in the case of decreasing material properties with increasing radius are shown in Figure 6a, Figure 6b, Figure 6c and Figure 6d, respectively.

The error between the analytical and finite element solutions in the multi-layer cylinder, in the case of decreasing material properties with increasing radius, is reported in Table 11.

## 4. Discussion

In this study, two cases of bi-layered and multi-layered thick-walled, functionally graded, hyperelastic cylinders subjected to internal pressure are investigated. The Mooney–Rivlin material model is used to account for the hyperelasticity and large deformation. Distinct material properties are assigned to each layer of the cylinder. The analytical formulation ensures the incorporation of incompressibility conditions, which is not guaranteed when using numerical software such as COMSOL. The continuity condition of the radial displacement is also defined as a constraint to ensure that its values at the shared edges are compatible with each other.

According to Figure 3, Figure 4, Figure 5 and Figure 6, the comparison between the distributions of radial displacement and stress components, including radial, tangential, and axial, derived from both the numerical and analytical solutions, shows excellent agreement, which indicates that the proposed analytical solution can properly predict the fields across the layers of the cylinder.

According to the second subfigures of Figure 3, Figure 4, Figure 5 and Figure 6, the inner layer, which is in direct contact with the applied pressure, demonstrates the highest amount of stress compared to the outer layers, which are significantly smaller due to their lower material properties. This behavior is observed in both the bi-layer and multi-layer cylinders, indicating that, regardless of the number of layers, more compliant materials lead to lower stress concentration. Both the tangential and axial stresses are tensile, which is typical for pressurized cylinders. Both stress components show discontinuities at the interfaces between layers due to the differences in material properties.

In this work, three values of 0.2, 0.15, and 0.1 MPa for internal pressure are investigated for each material gradation case. Regardless of the number of layers, excellent agreement between the analytical and FEM solutions, with errors below 1%, is recorded. As the internal pressure increases, the magnitude of the stress components follows the same trend and increase, and the errors increase slightly. This observation highlights the sensitivity of the error margin to higher internal pressures, but the discrepancies remain small and within an acceptable range.

The findings can provide valuable insights for designing FG pressurized cylinders, as the stress and displacement distributions can be optimized by selecting appropriate material properties across the thickness of the cylinder, which leads to more efficient and durable designs. The proposed closed-form solution can be used by engineers and designers to predict the behavior of multi-layer, functionally graded, hyperelastic cylinders under pressure, without the need for complex and time-consuming numerical simulations.

Engineers should also consider the material gradation to achieve a more uniform stress distribution, which is essential for the structural integrity of the vessels. Multi-layer cylinders with smoothly varying properties across the radius demonstrate reduced stress concentrations and improved overall performance when subjected to internal pressure.

The material distribution followed a deterministic approach: the inner layer properties were based on experimental data, while the middle and outer layers were established via fixed 30% and 50% inter-layer stiffness transitions. Continuous intra-layer gradation was then prescribed using 0.8 and 1.2 multiplication factors to demonstrate the model’s capacity for stress redistribution. This study provides a novel exact closed-form solution for such complex media, proving that continuous gradation reduces peak hoop stress and eliminates interfacial discontinuities compared to a discrete benchmark of identical material volume. The systematic optimization of these inter-layer drops and intra-layer factors represents a promising future outlook for this work.

The importance of the proposed exact formulation lies in its potential in rapid and iterative design. In engineering contexts, such as the development of biomimetic vascular grafts or elastomeric seals, designers often aim to minimize peak hoop stresses to prevent rupture. Because our solution is algebraic, it can be integrated into optimization algorithms in order to identify an optimum functional gradation for a specific operating pressure [44]. This “inverse design” capability allows for the creation of customized components that can maximize the resilience and minimize the volume of material used. This has a significant advantage over conventional numerical methods, which are based on trial and error in the initial stages of product development.

Future research could explore the addition of viscoelastic behavior, where time-dependent deformations and energy dissipation play an important role under cyclic loading conditions [45]. In order to further provide insight into this prediction, incorporation of damage mechanics into the model could be a promising approach for failure prediction particularly when fatigue or localized material degradation are concerns for designers [46,47]. Additionally, the use of shape memory behavior could be an interesting avenue for future work, particularly in systems where self-healing or adaptive responses to external loads are desired.

Although this study presents an exact mathematical foundation through an analytical solution, the authors acknowledge that experimental validation is a crucial step for industrial implementation. The current framework serves as a “ground truth” for verification, ensuring numerical models remain free from artifacts like volumetric locking. Future research will focus on practical implementation using advanced manufacturing to fabricate continuously graded elastomers, which will allow for the direct measurement of pressure–deformation curves and validation of the stress redistribution benefits identified by this analytical theory.

## 5. Conclusions

This study presents an exact and closed-form solution for predicting the mechanical response of hyperelastic, thick-walled, and multi-layered cylinders made of PVC. By implementing the Mooney–Rivlin constitutive model along with an exponential–logarithmic gradation law, this formulation provides an exact framework for the analysis of large deformations caused by internal radial pressure ranging from 0.1 to 0.2 MPa. A key advantage of this analytical approach is the exact implementation of the incompressibility condition, which bypasses numerical instabilities such as volumetric locking, which is regularly observed in standard FEM software.

The results demonstrate that the proposed solution is highly accurate, as the predictions of displacement and stress in all bi-layered and tri-layered configurations yielded an error of less than 1% compared to the mixed-formulation finite element results. Significantly, the analysis has shown that continuous grading can be considered an effective technique for smoothing the distribution of stress and significantly reduces the peak hoop stresses and artificial discontinuities that occur at the interfaces of conventional discrete layers. Finally, this parameter-transparent model proposes a computationally efficient tool for the inverse design of high-performance components. This allows for the optimization of material gradients in biomedical and industrial pressure vessels before proceeding to costly numerical or experimental phases.

## Figures and Tables

**Table 1:** Mooney–Rivlin hyperelastic properties of PVC [37,38].

Material	$C_{10}$ [MPa]	$C_{01}$ [MPa]
PVC	1.4780	3.3150

**Table 2:** Mooney–Rivlin hyperelastic properties of the bi-layer cylinder **(MF: 1.2)**.

Layer	$C_{10,min}$ [MPa] (at *R_out_*)	$C_{10,max}$ [MPa] (at *R_in_*)	$C_{01,min}$ [MPa] (at *R_out_*)	$C_{01,max}$ [MPa] (at *R_in_*)
Inner	1.478	1.7736	3.315	3.978
Outer	0.7390	0.8868	1.6575	1.989

**Table 3:** Mooney–Rivlin hyperelastic properties of the bi-layer cylinder **(MF: 0.8)**.

Layer	$C_{10,max}$ [MPa] (at *R_out_*)	$C_{10,min}$ [MPa] (at *R_in_*)	$C_{01,max}$ [MPa] (at *R_out_*)	$C_{01,min}$ [MPa] (at *R_in_*)
Inner	1.478	1.1824	3.315	2.6520
Outer	0.7390	0.5912	1.6575	1.3260

**Table 4:** Mooney–Rivlin hyperelastic properties of the multi-layer cylinder **(MF: 1.2)**.

Layer	$C_{10,min}$ [MPa] (at *R_out_*)	$C_{10,max}$ [MPa] (at *R_in_*)	$C_{01,min}$ [MPa] (at *R_out_*)	$C_{01,max}$ [MPa] (at *R_in_*)
Inner	1.478	1.7736	3.315	3.978
Middle	1.0346	1.2415	2.3205	2.7864
Outer	0.5912	0.7094	1.3260	1.5912

**Table 5:** Mooney–Rivlin hyperelastic properties of the multi-layer cylinder **(MF: 0.8)**.

Layer	$C_{10,max}$ [MPa] (at *R_out_*)	$C_{10,min}$ [MPa] (at *R_in_*)	$C_{01,max}$ [MPa] (at *R_out_*)	$C_{01,min}$ [MPa] (at *R_in_*)
Inner	1.478	1.1824	3.315	2.6520
Middle	1.0346	0.8277	2.3205	1.8564
Outer	0.5912	0.4730	1.3260	1.0608

**Table 6:** The geometrical properties of the layers in the bi-layer hyperelastic cylinder.

Layer	Inner Radius [mm]	Outer Radius [mm]
Inner	5	7
Outer	7	9

**Table 7:** The geometrical properties of the layers in the multi-layer hyperelastic cylinder.

Layer	Inner Radius [mm]	Outer Radius [mm]
Inner	5	7
Middle	7	9
Outer	9	11

**Table 8:** The error between the ANL and FEM solutions in the bi-layer cylinder (increasing material properties with increasing radius).

Pressure [MPa]	Radial Displacement Error (%)	Radial Stress Error (%)	Tangential Stress Error (%)	Axial Stress Error (%)
0.2	0.64	0.38	0.13	0.19
0.15	0.48	0.35	0.10	0.16
0.1	0.31	0.33	0.08	0.14

**Table 9:** The error between the ANL and FEM solutions in the bi-layer cylinder (decreasing material properties with increasing radius).

Pressure [MPa]	Radial Displacement Error (%)	Radial Stress Error (%)	Tangential Stress Error (%)	Axial Stress Error (%)
0.2	0.97	0.43	0.19	0.25
0.15	0.72	0.39	0.14	0.21
0.1	0.47	0.35	0.10	0.17

**Table 10:** The error between the ANL and FEM solutions in the multi-layer cylinder (increasing material properties with increasing radius).

Pressure [MPa]	Radial Displacement Error (%)	Radial Stress Error (%)	Tangential Stress Error (%)	Axial Stress Error (%)
0.2	0.81	0.39	0.23	0.34
0.15	0.60	0.35	0.18	0.29
0.1	0.40	0.31	0.13	0.24

**Table 11:** The error between the ANL and FEM solutions in the multi-layer cylinder (decreasing material properties with increasing radius).

Pressure [MPa]	Radial Displacement Error (%)	Radial Stress Error (%)	Tangential Stress Error (%)	Axial Stress Error (%)
0.2	0.54	0.33	0.16	0.26
0.15	0.40	0.31	0.13	0.23
0.1	0.26	0.28	0.10	0.21

## Data Availability

The original contributions presented in this study are included in the article. Further inquiries can be directed to the corresponding authors.

## Abbreviations

The following abbreviations are used in this manuscript:

FG	Functionally Graded
PVC	Polyvinyl Chloride
FEM	Finite Element Method
ANL	Analytical
MF	Multiplication Factor

## Appendix A

The closed-form analytical solution obtained after integrating Equation (36), as expressed in Equation (A1), involves the parameters $M=\frac{C_{10_{max}}}{C_{10_{min}}}$, $N=\frac{C_{01_{max}}}{C_{01_{min}}}$, $C_{1}=C_{10_{max}}$, $C_{2}=C_{10_{min}}$, $C_{3}=C_{01_{max}}$, $C_{4}=C_{01_{min}}$, with $a=R_{in}$, $b=R_{out}$, and c representing the integration constant.

(A1)
$$\begin{array}{l} \frac{\left(C_{3}\ln^{2}(N)+C_{1}\ln^{2}(M))cb\ln(|R^{2}+c|\right)}{(b-a{)}^{3}}-\frac{\left(C_{3}\ln^{3}(N)+C_{3}\ln^{2}(N)+C_{1}\ln^{3}(M)+C_{1}\ln^{2}(M))ca\ln(|R^{2}+c|\right)}{(b-a{)}^{3}}-\frac{\left(C_{3}+C_{1})b^{3}\ln(|R^{2}+c|\right)}{(b-a{)}^{3}}\\ \;+\frac{\left(C_{3}\ln(N)+C_{1}\ln(M)+3C_{3}+3C_{1})ab^{2}\ln(|R^{2}+c|\right)}{(b-a{)}^{3}}\\ \;-\frac{\left(C_{3}\ln^{2}(N)+4C_{3}\ln(N)+C_{1}\ln^{2}(M)+4C_{1}\ln(M)+6C_{3}+6C_{1})a^{2}b\ln(|R^{2}+c|\right)}{(b-a{)}^{3}}\\ \;+\frac{\left(C_{3}\ln^{3}(N)+3C_{3}\ln^{2}(N)+6C_{3}\ln(N)+6C_{3})a^{3}\ln(|R^{2}+c|\right)}{6(b-a{)}^{3}}+\frac{\left(C_{1}\ln^{3}(M)+3C_{1}\ln^{2}(M)+6C_{1}\ln(M)+6C_{1})a^{3}\ln(|R^{2}+c|\right)}{6(b-a{)}^{3}}\\ \;+\frac{2((C_{3}+C_{1})b^{3}+(C_{3}\ln(N)+C_{1}\ln(M)-3C_{3}-3C_{1})ab^{2})\ln(|R|)}{(b-a{)}^{3}}\\ \;-\frac{\left(C_{3}\ln^{2}(N)+4C_{3}\ln(N)+C_{1}\ln^{2}(M)+4C_{1}\ln(M)+6C_{3}+6C_{1})a^{2}b\ln(|R|\right)}{(b-a{)}^{3}}\\ \;+\frac{\left(C_{3}\ln^{3}(N)+3C_{3}\ln^{2}(N)+6C_{3}\ln(N)+6C_{3})a^{3}\ln(|R|\right)}{3(b-a{)}^{3}}+\frac{\left(C_{1}\ln^{3}(M)+3C_{1}\ln^{2}(M)+6C_{1}\ln(M)+6C_{1})a^{3}\ln(|R|\right)}{3(b-a{)}^{3}}\\ \;-\frac{\left(5C_{3}\ln^{3}(N)+5C_{1}\ln^{3}(M))c^{3}\tan^{-1}(\frac{R}{\sqrt{c}}\right)}{6(b-a{)}^{3}\sqrt{c}}+\frac{\left(3C_{3}\ln(N)+3C_{1}\ln(M))b^{2}c\tan^{-1}(\frac{R}{\sqrt{c}}\right)}{(b-a{)}^{3}\sqrt{c}}\\ \;-\frac{\left(3C_{3}\ln^{2}(N)+6C_{3}\ln(N)+3C_{1}\ln^{2}(M)+6C_{1}\ln(M))abc\tan^{-1}(\frac{R}{\sqrt{c}}\right)}{(b-a{)}^{3}\sqrt{c}}+\frac{\left(3C_{3}\ln^{3}(N)+6C_{3}\ln^{2}(N)+6C_{3}\ln(N))a^{2}c\tan^{-1}(\frac{R}{\sqrt{c}}\right)}{(b-a{)}^{3}\sqrt{c}}\\ \;+\frac{\left(3C_{1}\ln^{3}(M)+6C_{1}\ln^{2}(M)+6C_{1}\ln(M))a^{2}c\tan^{-1}(\frac{R}{\sqrt{c}}\right)}{(b-a{)}^{3}\sqrt{c}}+\frac{(C_{3}\ln^{3}(N)+C_{1}\ln^{3}(M))c^{2}R-6(C_{3}\ln(N)+C_{1}\ln(M))b^{2}cR}{6(b-a{)}^{3}R^{2}+6(b-a{)}^{3}c}\\ \;+\frac{(C_{3}\ln^{2}(N)+2C_{3}\ln(N)+C_{1}\ln^{2}(M)+2C_{1}\ln(M))abcR}{(b-a{)}^{3}R^{2}+(b-a{)}^{3}c}\\ \;-\frac{(C_{3}\ln^{3}(N)+2C_{3}\ln^{2}(N)+2C_{3}\ln(N)+C_{1}\ln^{3}(M)+2C_{1}\ln^{2}(M)+2C_{1}\ln(M))a^{2}cR}{2(b-a{)}^{3}R^{2}+2(b-a{)}^{3}c}\\ \;+\frac{((C_{3}\ln^{2}(N)+C_{1}\ln^{2}(M))b-(C_{3}\ln^{3}(N)+C_{3}\ln^{2}(N)+C_{1}\ln^{3}(M)+C_{1}\ln^{2}(M))a)c^{2}}{2(b-a{)}^{3}R^{2}+2(b-a{)}^{3}c}\\ \;+\frac{((-C_{3}-C_{1})b^{3}c+(C_{3}\ln(N)+C_{1}\ln(M)+3C_{3}+3C_{1})ab^{2}c}{(b-a{)}^{3}R^{2}+(b-a{)}^{3}c}\\ \;-\frac{(C_{3}\ln^{2}(N)+4C_{3}\ln(N)+C_{1}\ln^{2}(M)+4C_{1}\ln(M)+6C_{3}+6C_{1})a^{2}bc}{2(b-a{)}^{3}R^{2}+2(b-a{)}^{3}c}\\ \;+\frac{(C_{3}\ln^{3}(N)+3C_{3}\ln^{2}(N)+6C_{3}\ln(N)+C_{1}\ln^{3}(M)+3C_{1}\ln^{2}(M)+6C_{1}\ln(M)+6C_{3}+6C_{1})a^{3}c}{6(b-a{)}^{3}R^{2}+6(b-a{)}^{3}c}+\frac{2(C_{3}\ln^{3}(N)+C_{1}\ln^{3}(M))cR}{3(b-a{)}^{3}} \end{array}$$
